# Genotyping of Endosperms to Determine Seed Dormancy Genes Regulating Germination Through Embryonic, Endospermic, or Maternal Tissues in Rice

**DOI:** 10.1534/g3.114.015362

**Published:** 2014-12-04

**Authors:** Xing-You Gu, Jinfeng Zhang, Heng Ye, Lihua Zhang, Jiuhuan Feng

**Affiliations:** Plant Science Department, South Dakota State University, Brookings, South Dakota 57007

**Keywords:** seed dormancy, quantitative trait locus, endosperm, segregation distortion, rice

## Abstract

Seed dormancy is imposed by one or more of the embryo, endosperm, and maternal tissues that belong to two generations and represent two ploidy levels. Many quantitative trait loci (QTL) have been identified for seed dormancy as measured by gross effects on reduced germination rate or delayed germination in crop or model plants. This research developed an endosperm genotype−based genetic approach to determine specific tissues through which a mapped QTL regulates germination using rice as a model. This approach involves testing germination velocity for partially after-ripened seeds harvested from single plants heterozygous for a tested QTL and genotyping endosperms from individual germinated and nongerminated seeds with a codominant DNA marker located on the QTL peak region. Information collected about the QTL includes genotypic frequencies in germinated and/or nongerminated subpopulations; allelic frequency distributions during a germination period; endosperm or embryo genotypic differences in germination velocity; and genotypic frequencies for gametes involved in the double fertilization to form the sampled seeds. Using this approach, the seed dormancy loci *SD_12_*, *SD_1-2_*, and *SD_7-1_* were determined to regulate germination through the embryo, endosperm, and maternal tissues, respectively; *SD_12_* and *SD_1-2_* acted additively on germination velocity in the offspring tissues; and *SD_12_* also was associated with the preferential fertilization of male gametes in rice. This new genetic approach can be used to characterize mapped genes/QTL for tissue-specific functions in endospermic seeds and for marker-assisted selection of QTL alleles before or immediately after germination in crop breeding.

Seed dormancy developed on the mother plant (*i.e.*, primary dormancy) is imposed by one or more of the embryo, endosperm, and maternal tissues that belong to two (maternal and offspring) generations and represent two (diploid and triploid) ploidy levels. Genotypic variation of seed dormancy exits in natural populations and crop germplasm as an adaptive mechanism for seed-bearing plants to regulate the timing of germination. The natural variation in seed dormancy or germination-related traits has been associated with multiple quantitative trait loci (QTL) in barley (*e.g.*, Ullrich *et al.* 1993), wheat (*e.g.*, Anderson *et al.* 1993), rice (*e.g.*, Lin *et al.* 1998), oats (Fennimore *et al.* 1999), sorghum (*e.g.*, Lijavetzky *et al.* 2000), Arabidopsis (*e.g.*, Alonso-Blanco *et al.* 2003), lettuce (*e.g.*, Argyris *et al.* 2005), sunflower (*e.g.*, Gandhi *et al.* 2005), rye (Masojć *et al.* 2007), oilseed rape (Schatzki *et al.* 2013), and peach (Blaker *et al.* 2013). Of the QTL reported in the listed and other research, only those in peach were detected based on marker-trait associations in the same generation (*i.e.*, F_2_ seeds, Blaker *et al.* 2013); the remaining loci in all other species were claimed based on associations between plant genotypes and germination capabilities of seeds from the plants in mapping populations, which ignored the difference in generation or genotype between seed component tissues. Technically, it is difficult to genotype both plants and individual seeds from the plants in a mapping population. Statistical models, which combine maternal with offspring (embryo or endosperm) genotypes to map QTL associated with seed traits (*e.g.*, Zhang *et al.* 2004), have not been used for research on seed dormancy. Thus, additional research is needed to determine specific tissues in which a QTL underlying gene is expressed to impose seed dormancy. This information is critical to further understand cellular and molecular mechanisms of seed dormancy and is also useful to develop selection strategies to manipulate germinability (*e.g.*, the resistance to preharvest sprouting in cereal crops) in breeding programs using the QTL alleles.

Experimental approaches that have been used to infer the involvement of a seed component tissue in dormancy imposition can be grouped into somatic, molecular and genetic categories. Somatic approaches have to resort to exercising embryos or physically removing the maternal tissues testa and pericarp (also known as seed and fruit coats) covering caryopses in grass species. The isolated embryos or naked caryopses were germinated on a selected medium to infer the presence of embryo or coat-imposed dormancy, as did in Arabidopsis, barley, oat, rice, and wheat (Takahashi 1963; Morris *et al.* 1989; Foley 1992; Wang *et al.* 1995; Lee *et al.* 2010). Molecular approaches include various methods for gene tissue-specific expression analyses, such as RNA *in situ* hybridization and *GUS* reporter assay, which require DNA/mRNA sequences of a known gene to prepare probes (vectors) for hybridization (transformation). Via the use of molecular approaches, genes cloned from the rice *qLTG3-1* and *Sdr4* and the wheat *QPhs.ocs-3A1* (*TaMFT*) QTL were implicated to regulate dormancy or low-temperature germination in embryos (Fujino *et al.* 2008; Sugimoto *et al.* 2010; Nakamura *et al.* 2011). Genetic approaches start with crosses between genotypes different in seed dormancy or QTL allele to obtain hybrid F_1_ or F_2_ seeds. F_1_ seed samples from reciprocal crosses were tested for the difference in germinability to infer a maternal tissue effect on seed dormancy, as reported for rice and wheat (Nair *et al.* 1965; Noll *et al.* 1982; Flintham 2000). The F_2_ seed samples were partially after-ripened to test embryo genotypic frequencies for a QTL marker in the germinated and/or nongerminated subpopulations; the QTL was inferred to be involved in the maternal tissue-imposed dormancy if the genotypic/allelic frequencies kept constant between the subpopulations, or involved in the offspring tissue-imposed dormancy if the dormancy-reducing allele had a greater frequency in the germinated than in the nongerminated subpopulation (Gu *et al.* 2008). By use of the embryo genotype-based genetic approach, the *qSD7-1* and *qSD12* QTL were associated with maternal and offspring tissue-imposed dormancies, respectively, in rice.

Advantages of the genetic over the somatic and molecular approaches are nondestructive testing and applicability to a large sample of seeds. Concerns about the genetic approaches are some uncertainties in inferences for tissue specificity or about interference by a linked segregation distortion locus (SDL; Gu *et al.* 2008). First, a maternal tissue effect on germination inferred by the difference between reciprocal F_1_s may be confounded with a cytoplasmic or endospermic effect, because hybrid seeds have female cytoplasm in all their cells and two of the three chromosomal sets in an endosperm cell from the female parent. The uncertainty about a cytoplasmic effect can be resolved by the embryo genotype-based genetic approach, as F_2_ seeds self-pollinated from an F_1_ plant are identical in cytoplasm. Second, the embryo genotype-based approach cannot distinguish an embryonic from an endospermic effect. An embryo is developed from a fertilized egg (2n) fused between the egg cell (n) in an embryo sac and one of the two sperms (n) in a pollen tube, whereas the endosperm is developed from the primary endosperm nucleus (3n) fused between two genetically identical polar nuclei (n) in the sac and the other sperm. Thus, from an F_1_ plant heterozygous for dormancy locus (*D/d*), the F_2_ seeds with the *Dd* genotype embryo have either *DDd* or *Ddd* genotype endosperm. Theoretically, if the dormancy gene expresses in the triploid tissue, the endospermic effect, which could be confounded with the maternal tissue effect as inferred by the reciprocal F_1_ approach or cannot be detected by the embryo genotype-based approach, could be estimated by the association between germination velocity of individual F_2_ seeds and their endosperm genotypes. In case that the gene expresses in the diploid offspring tissue, the endosperm genotypes can be transformed into embryo genotypes to estimate the embryonic effect. Third, genotypic frequencies for a dormancy gene in a germinated or nongerminated subpopulation of partially after-ripened F_2_ seeds also can be affected by a linked SDL. An SDL was often associated with preferential dysfunction/fertilization of the male or female gamete (Lyttle 1991). Fortunately, gamete genotypic frequencies of an SDL for both male and female sides can be estimated based on frequencies of the four endosperm (but not the three embryo) genotypes in a random sample of F_2_ seeds. The estimates can help determine if a distorted segregation observed in a germinated or nongerminated subpopulation arises from the selection for less or more dormancy seeds, a linked SDL, or both.

This research was devoted to develop an endosperm genotype-based genetic approach to characterize functions of previously mapped seed dormancy QTL in regulating germination through the embryo, endosperm, or maternal tissues. Genetic and seed biology principles for the new approach are same as those described for the embryo genotype-based approach (Gu *et al.* 2008), except that genotyping information is collected from endosperms, rather than embryos. It is relatively easy to genotype embryos, because DNA samples for the genotyping can be prepared from embryonic leaves or seedlings after a germination test and there are only three embryo genotypes for a locus in an F_2_ seed population of a diploid species. Thus, challenges to this research would be techniques used to extract endospermic DNAs from individual seeds without a negative impact on standard germination testing and to display all four endosperm genotypes for a locus in an F_2_ seed population. In this research, rice (*Oryza sativa* L.) was used as a model to develop a marker-genotyping system to meet the technical challenges, and the seed dormancy QTL *SD_1-2_*, *SD_7-1_*, and *SD_12_*, which were isolated as single Mendelian factors into the same genetic background in the previous research, were selected to demonstrate the efficacy of the new genetic approach. This article summarized techniques and analytic strategies for the endosperm genotype-based genetic approach, presented new information about the three selected loci, and discussed implications of some discoveries from this research.

## Materials and Methods

### Parental lines and F_2_ seed populations

#### Isogenic lines (ILs):

Four ILs, including the recipient parent EM93-1, were used to develop hybrid F_1_s that are heterozygous for each of the *SD_1-2_*, *SD_7-1_*, and *SD_12_* loci or both *SD_7-1_* and *SD_12_* (Table 1). EM93-1 is a line of cultivated rice (*Oryza sativa* subsp. *indica*) with a semi-dwarf plant height controlled by the *semidwarf1* (*sd_1_*) gene and is homozygous for the dormancy-enhancing allele at *SD_1-2_* and the dormancy-reducing alleles at both *SD_7-1_* and *SD_12_*. The ILs were developed by introducing single chromosome (chr) segments from SS18-2, a line of wild-like weedy rice (*O*. *sativa*), into the recipient genetic background. Specifically, IL_sd1-2_ has an introgression segment of ~3000 kb in physical length containing both *Sd_1_* and *sd_1-2_* on chr 1 (Ye *et al.* 2013); IL_SD7-1_ has an introgression segment of ~2 kb intragenic to the pleiotropic gene *SD_7-1_*/*Rc* on chr 7 and the introgression converts the mutant allele in EM93-1 (*sd_7-1_*/*rc*) into a functional allele for both seed dormancy and red pericarp color (Gu *et al.* 2011); and IL_SD12_ has an introgression segment of ~200 kb containing the *SD_12_* allele on chr 12 (Gu *et al.* 2010).

**Table 1:** Genotypic and phenotypic information about isogenic lines (IL) and hybrid F_1_s used to develop F_2_ seed populations

Parental Line or F_1_*^a^*	Genotype*^a^*	Segment/Marker*^b^*	Pericarp Color*^c^*	Plant Height*^d^*
A. IL_SD1-2_ (EM93-1)	*SD_1-2_SD_1-2_sd_7-1_sd_7-1_sd_12_sd_12_*	Recipient	White (*rcrc*)	Semidwarf (*sd1sd1*, ~80 cm)
B. IL_sd1-2_	*sd_1-2_sd_1-2_sd_7-1_sd_7-1_sd_12_sd_12_*	~3000 kb	White (*rcrc*)	Tall (*Sd1Sd1*, ~100 cm)
C. IL_SD7-1_	*SD_1-2_SD_1-2_SD_7-1_SD_7-1_sd_12_sd_12_*	~2 kb	Red (*RcRc*)	Semidwarf (*sd1sd1*)
D. IL_SD12_	*SD_1-2_SD_1-2_sd_7-1_sd_7-1_SD_12_SD_12_*	~200 kb	White (*rcrc*)	Semidwarf (*sd1sd1*)
F_1_SD1-2_ (A×B)	*SD_1-2_sd_1-2_sd_7-1_sd_7-1_sd_12_sd_12_*	RM315/3602	White (*rcrc*)	Tall (*Sd1sd1*, **~**90 cm)
F_1_SD7-1_ (A×C)	*SD_1-2_SD_1-2_SD_7-1_sd_7-1_sd_12_sd_12_*	RID12	Red (*Rcrc*)	Semidwarf (*sd1sd1*)
F_1_SD12_ (A×D)	*SD_1-2_SD_1-2_sd_7-1_sd_7-1_SD_12_sd_12_*	SD12m13	White (*rcrc*)	Semidwarf (*sd1sd1*)
F_1_SD7-1SD12_ (C×D)	*SD_1-2_SD_1-2_SD_7-1_sd_7-1_SD_12_sd_12_*	RID12 and SD12m13	Red (*Rcrc*)	Semidwarf (*sd1sd1*)

a EM93-1 was the recipient of single introgression segments from a line of “red” weedy rice in ILs for the *SD_1-2_*, *SD_7-1_* or *SD_12_* locus. Upper or lower case letters indicate dormancy-enhancing (*SD*) or -reducing (*sd*) alleles at the three loci.

b Physical lengths of the introgression segments in kilobases or DNA markers selected to tag the loci.

c *Rc*, red pericarp color gene, which belongs to the same locus as *SD_7-1_*, with the functional allele responsible for both red pigment and enhanced seed dormancy (Gu *et al.* 2011).

d *sd1*, *semidwarf1* gene located on the *SD_1-2_*-containing region, with the EM93-1−derived allele responsible for both reduced plant height and enhanced seed dormancy (Ye *et al.* 2013).

#### F_2_ seed populations:

About 20 F_1_ hybrid plants from each of the four crosses were grown in a greenhouse, verified for genotypes at *SD_1-2_*, *SD_7-1_*, and *SD_12_* using markers on the introgression segments (Table 1), and self-pollinated to produce F_2_ seed populations. Seeds were harvested from individual F_1_ plants at 40 d after flowering, air-dried at the greenhouse for 3 d, and stored at a freezer (−20°) to maintain the primary dormancy.

### Seed after-ripening, germination, and subpopulatons

Seed samples from the freezer were after-ripened at the room temperature (24−25°) for 0−35 d to release part of the primary dormancy prior to germination testing. The time period of a partially after-ripening treatment varied with seed populations and was determined in preliminary experiments to manipulate relative sizes of germinated and nongerminates subpopulations. Two or more independent germination experiments were conducted for each of the four F_2_ populations and an experiment consisted of 600−2000 fully developed seeds. About 60 seeds derived from an F_1_ plant were distributed in a 9-cm Petri dish lined with a filter paper, soaked with 8 mL of deionized water, and incubated at 30° and 100% relative humidity in dark. Germination (radicle emerged >3 mm) counting started at 48 hr after imbibition and continued every 12 or 24 hr for 7 or ~10 d. Germinated seeds were transferred to new Petri dishes to collect the endosperm tissue. All germinated seeds from an experiment were formed a germinated subpopulation, whereas seeds that did not germinate and were not contaminated within 7 or 10 d were grouped as a nongerminated subpopulation.

### DNA microextraction and marker genotyping

For a germinated subpopulation, a newly germinated intact seed (spikelet) was cut in a cross section at 1/3 to the endosperm end. The embryo-less portion was cleaned by removing the maternal tissues and the endosperm tissue transferred into a 1.5-mL centrifuge tube and stored in a −20° freezer for DNA extraction. This method was also used to sample the endosperm tissue from nongerminated or dry seeds. In preliminary experiments to genotype both endosperm and embryo tissues, the sectioned seeds with an emerged radicle were transferred to 24-well cell culture plates (Corning 15.6-mm diameter) lined with wetted filter papers and placed in an illuminated growth chamber for several days to collect embryonic leaves for DNA extraction.

Endospermic DNA was extracted using methods modified from Chunwongse *et al.* (1993) and Kang *et al.* (1998). Primarily, the endosperm tissue was incubated in 200 µL of of lysis buffer (0.5% sodium dodecyl sulfate and 20 µg of Proteinase K) at 37° for 1 hr and ground with a spatula. The crude sample was mixed with 400 µL of 2% CTAB solution [2% (w/v) cetyltrimethylammonium bromide, 100 mM Tris-HCl (pH 8.0), 20 mM ethylenediaminetetraacetic acid (pH 8.0), 1.4 M NaCl, and 1% polyvinylpyrrolidone] and phase-separated with the 24:1 ratio of chloroform:isoamyl alcohol mixture. DNA in the aqueous phase was precipitated with cold isopropanol and washed with 70% ethanol. Air-dried DNA was dissolved in 50 µL of TE buffer (10 mM Tris-HCl and 1 mM EDTA at pH 8.0) and treated with 1 µL of RNaseA (10 mg/µL) to remove RNA. Alternatively, endosperm samples were placed in a 96-well plate and heated in 80 µL of lysis buffer (0.1 M NaOH) on a Thermocycler set at 95° for 15 min. The lysing solutions were mixed with 80 µL of neutralization buffer (10 mM Tris-HCl and 0.1 M HCl at pH 2.0) and the supernatant was used as DNA templates for polymerase chain reaction (PCR). DNA extraction from the embryonic leaves was conducted using the previously described method (Gu *et al.* 2008).

The simple sequence repeat or insertion/deletion markers on partial high-resolution maps for the *SD_1-2_* (Ye *et al.* 2013), *SD_7-1_* (Gu *et al.* 2011), or *SD_12_* (Gu *et al.* 2010) regions were selected to optimize a genotyping system. The selected markers are codominant, different in size by 10 or more bp between the two alleles, and capable of distinguishing four endosperm genotypes of F_2_ seeds by regular PCR and gel electrophoresis. The PCR amplification was performed using a 20-μL volume containing 40 ng of DNA template, 4 µL of 5× Green GoTaq reaction buffer (Promaga), 200 µM dNTP, 50 nM each primer, and 2 units of Taq polymerase, in a BIO-RAD Thermocycler. The PCR program was initiated at 94° for 5 min, followed by 35 cycles of denature at 94° for 30 sec, annealing at 55° for 30 sec and elongation at 72° for 45 sec, and ended with a final elongation at 72° for 7 min. PCR products were separated in a 6% nondenaturing polyacrylamide gel at ~300 V for ~3 hr and displayed and recorded using the AlphaEaseFC (Alpha Innotech) gel imaging system.

### Data analysis and genetic inferences

#### Genotypic and allelic frequencies:

Seeds (F_2_) self-pollinated from a hybrid F_1_ plant are identical for the maternal tissue genotype (*Dd*), but vary in embryo (*DD*, *Dd*, and *dd*) and endosperm (*DDD*, *DDd*, *Ddd*, and *ddd*) genotypes for a locus with two alleles that enhance (*D*) or reduce (*d*) seed dormancy (Table 2). The endosperm genotypic frequencies *F_DDD_*, *F_DDd_*, *F_Ddd_*, and *F_ddd_* in a subpopulation or a random sample were calculated as described in Table 2 and used to estimate the overall allelic frequencies *F_D_* and *F_d_* in the sample and the genotypic frequencies for male (*F_D_*_’_ and *F_d_*_’_) and female (*F_D’_*_’_ and *F_d’_*_’_) gametes involved in the double fertilization to develop the F_2_ seeds. The observed genotypic and allelic frequencies may or may not follow Hardy-Weinberg Equilibrium, depending on responses of the dormancy gene to the selection for germinated or nongerminated subpopulations and/or the presence or absence of a SDL in the gene-containing region (Gu *et al.* 2008). Thus, χ^2^ testing was used to determine the fitness of endosperm genotypic frequencies to the 0.25:0.25:0.25:0.25 expectation and binomial testing used to determine the fitness of allelic and gamete genotypic frequencies to the 0.5 expectation. Genetic inferences from the statistical tests were: 1) the gene was involved in offspring tissue-imposed dormancy and responded to the selection when genotypic frequencies deviate from the expectations with *F_d_* greater in the germinated than in the nongerminated subpopulation; 2) the gene was involved in maternal tissue-imposed dormancy and did not responded to the selection when genotypic frequencies fit the expectations with *F_d_* constant across subpopulations; and 3) there is a SDL in the dormancy gene-containing region when genotypic frequencies deviate from the expectations in the joined population of germinated and nongerminated seeds or in a random sample.

**Table 2:** List of genotypes for seed component tissues and endosperm genotypic frequencies in an F_2_ seed population segregating for a dormancy locus (*D/d*)

Female Gamete	Male Gamete		Genotypic Frequency*^a^*
	*d* (0.5)	*D* (0.5)	
*d* (0.5)*^b^*	*ddd* (*dd*) [*Dd*]*^c^*	*Ddd* (*Dd*) [*Dd*]	*F_d’_*_’_ = *F_ddd_* + *F_Ddd_*
	*F_ddd_* = N*_ddd_*/N (0.25)*^b^*	*F_Ddd_* = N*_Ddd_*/N (0.25)	
*D* (0.5)	*DDd* (*Dd*) [*Dd*]	*DDD* (*DD*) [*Dd*]	*F_D’_*_’_ = *F_DDd_* + *F_DDD_*
	*F_DDd_* = N*_DDd_*/N (0.25)	*F_DDD_* = N*_DDD_*/N (0.25)	
Genotypic frequency*^a^*	*F_d_*_’_ = *F_ddd_* + *F_DDd_*	*F_D_*_’_ = *F_Ddd_* + *F_DDD_*	
Overall allelic frequency	*F_d_ = F_ddd_* + 0.5*F_Ddd_* + 0.5*F_DDd_*	*F_D_ = F_DDD_* + 0.5*F_DDd_* + 0.5*F_Ddd_*	

a Genotypic frequencies for male (*F_d_*_’_ and *F_D_*_’_) and female (*F_d’_*_’_ and *F_D’_*_’_) gametes involved in the double fertilization to form the seed population.

b The value in the parentheses is the Mendelian expectation for the endosperm (0.25) or gamete (0.5) genotypic frequency, or the overall allelic frequency (0.5).

c Genotypes for the endosperm (triploid), embryo (parentheses), and maternal (brackets) tissues of a seed at a dormancy locus with the two functionally differentiated alleles *D* and *d*. *F_ddd_*, *F_Ddd_*, *F_DDd_*, and *F_DDD_* are genotypic frequencies for the *ddd*, *Ddd*, *DDd*, and *DDD* endosperms, respectively, estimated based on the number of seeds for individual genotypes (N*_ddd_*, N*_Ddd_*, N*_DDd_*, and N*_DDD_*) and the population size (N).

Binomial testing also was used to determine the equity or difference in genotypic frequency between male and female gametes (*e.g.*, *F_d_*_’_ = *F_d’_*_’_). SE used for a test was calculated as (2*F_d_* × *F_D_*/N)^1/2^, where N is the number of genotyped seeds in a subpopulation or random sample. Significant differences in the tests were used to infer underlying mechanisms, such as a dosage effect of the dormancy gene on germination velocity in endosperms (*F_d’_*_’_ > *F_d_*_’_), or a differentiation of the SDL in preferential fertilization between male and female gametes (*F_d_*_’_ > *F_d’_*_’_).

#### Germination distributions:

A sample of partially after-ripened seeds is characterized by germination heterogeneity, *i.e.*, some germinate earlier than others and some dormant seeds never germinate in an experiment. The heterogeneity occurs in a sample of seeds from a pure line (due to nongenetic factors) and a segregating (*e.g.*, F_2_) population (due to both genetic and nongenetic factors). To quantify the heterogeneity in an experiment, the daily counted germination data from all samples were used to develop a germination distribution:

$$y_{j}=\sum_{j}N_{gj}/N\left(100\%\right)$$ (1)

where, *y_j_* is the cumulative germination rate at day *j* (*j* = 2 to 7 or 10), *Σ_j_N_gj_* is the summation of seeds germinated from day 2 to day *j*, and *N* is the total number of seeds tested in the experiment. The germination distribution was compared with allelic frequency distributions in the germinated subpopulation to infer whether the dormancy gene is involved in the regulation of germination through the offspring or maternal tissues.

For experiments genotyped for both germinated and nongerminated seeds, the germination distribution was calculated for individual endosperm genotypes:

$$y_{ij}=\sum_{j}N_{gij}/\left(\sum_{j}N_{gij}+N_{ng}\cdot F_{ngi}\right)\left(100\%\right)$$ (2)

where, *y_ij_* is the cumulative germination percentage for the *i*th endosperm genotype (*i* = 0, 1, 2, and 3, the copy number of *D* allele in the genotype) on day *j*; *Σ_j_N_gij_* is the summation of the *i*th genotypic seeds germinated from days 2 to *j*; *N_ng_* is the total number of nongerminated seeds (including those with missing genotyping data) in the experiment; and *F_ngi_* is the frequency of genotype *i* in the nongerminated subpopulation. The difference in germination distribution pattern among the genotypes was used to infer whether the gene is involved in the genetic control of seed dormancy imposed by the embryo, endosperm or maternal tissues.

#### Genetic effect estimation:

For genes involved in the regulation of germination through the embryo or endosperm tissue, their component effects in germinated subpopulations were estimated using linear regression models for embryo genotypes:

$$y_{ik}=\mu +ax_{i}+dz_{i}+\varepsilon_{ik}$$ (3)

or for endosperm genotypes (Mo 1988):

$$y_{ik}=\mu +ax_{i}+d_{1}z_{1i}+d_{2}z_{2i}+\varepsilon_{ik}$$ (4)

where: *y_ik_* is the incubation time required to complete germination for seed *k* (*k* = 1 to *Ng*, the number of genotyped germinated seeds) of the *i*th endosperm genotype; *µ* is the model mean; *x_i_* is the dummy variable for the additive component and is coded as −1, 0, and 1 for the embryo genotypes *dd*, *Dd*, and *DD*, respectively, or coded as −1.5, −0.5, 0.5, and 1.5 for the endosperm genotypes *ddd*, *Ddd*, *DDd*, and *DDD*, respectively; *z_i_* is the dummy variable for the dominance component and is coded as −0.5 for both *dd* and *DD* or 0.5 for *Dd*; *z_1i_* is the dummy variable for the first dominance component of the two *D* alleles over the *d* allele in the *DDd* genotype and is coded as 1 for *DDd* or 0 for the remaining three endosperm genotypes; *z_2i_* is the dummy variable for the second dominance component of the *D* allele over the two *d* alleles in the *Ddd* genotype and is coded as 1 for *Ddd* or 0 for the remaining three endosperm genotypes; *a*, *d*, *d_1_*, and *d_2_* are partial regression coefficients for corresponding variables and the estimates of gene additive or dominance effects; and *ε_ik_* is the residual effect of the model. Regression analysis was implemented using the REG procedure of SAS 9.3 (SAS Institute 2011) with a stepwise selection set at the significance level of probability <5%.

For the germinated subpopulation segregating for both *SD_7-1_* and *SD_12_*, two-way analysis of variance was used to detect their main and interactional effects on germination velocity. The variance analysis was conducted based on embryo genotypes using the two-factor factorial model:

$$y_{ijk}=\mu +\alpha_{i}+\beta_{j}+{\left(\alpha \beta \right)}_{ij}+\varepsilon_{ijk}$$ (5)

where, *y_ijk_* is the incubation time required to complete germination for the *k*th seed having the *i*th genotype at *SD_7-1_* and the *j*th genotype at *SD_12_*, *µ* is the model mean; *α_i_* and *β_j_* are main effects of *SD_7-1_* and *SD_12_*, respectively; (*αβ*)_ij_ is the interactional effect between the two loci; and *ε_ijk_* is the error term of the model.

## Results

### Reliability of the marker genotyping system to distinguish endospermic genotypes

**Figure 1:**
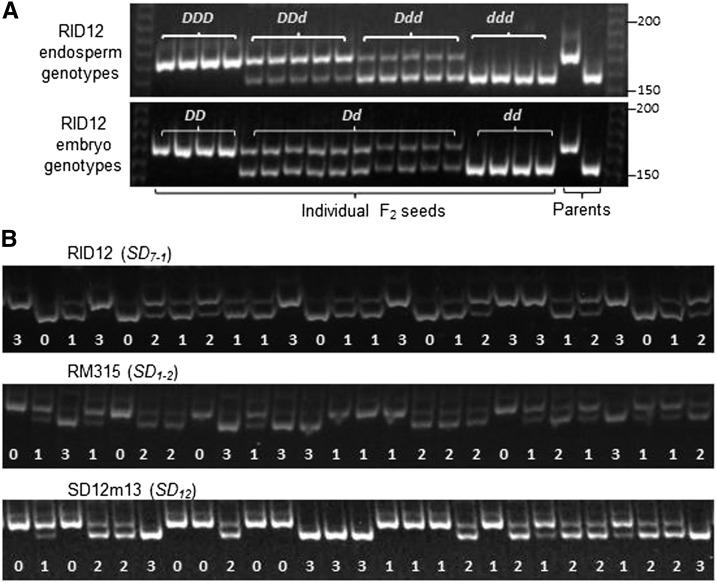
Electrophoresis patterns of endosperm genotypes for codominant markers. (A) Comparison between endosperm and embryo genotypes of same seeds. (B) Segregation patterns for four endosperm genotypes of F_2_ seeds. RID12, RM315, and SD12m13 were selected to mark the seed dormancy loci *SD_7-1_*, *SD_1-2_*, and *SD_12_*, respectively. Gel images show electrophoresis patterns for individual F_2_ seeds from germinated subpopulations. The genotypes are indicated by combinations of the dormancy-enhancing (*D*) and/or -reducing (*d*) alleles, or by the copy number of the *D* allele (0−3) at a locus.

The markers RID12, RM315/3602, and SD12m13, which were selected to tag *SD_7-1_*, *SD_1-2_*, and *SD_12_*, respectively, could separate both embryo and endosperm genotypes in F_2_ seed populations. As shown on gel images for RID12 (Figure 1A), the two homozygous (*DDD* and *ddd*) endosperms displayed one of the two alleles (bands), which are same as the homozygous (*DD* and *dd*) embryos; whereas the two heterozygotes (*DDd* and *Ddd*) showed both bands (codominance) with one brighter than the other (dosage effect), which are different from the heterozygous (*Dd*) embryos that have the two bands equal in signal intensity. All the selected markers were consistent in gel image patterns (*i.e.*, codominance and dosage effect) in F_2_ seed populations (Figure 1B). Of the 11 germination experiments, 9 had >90% germinated or nongerminated seeds genotyped with this marker-genotyping system, and the success rate was similar to that (93%) for a random sample of seeds segregating for *SD_12_* (Table 3). These results demonstrated that the quality of endospermic DNAs from germinated seeds is good for genotyping with the PCR-based markers and the genotypes are readily converted into embryo genotypes.

**Table 3:** Summary of genotypic and allelic frequencies for the *SD_7-1_*, *SD_1-2_*, or *SD_12_* locus in F_2_ seed subpopulations or joined populations

Experiment*^a^*	Subpopulation (Genotyped Seeds)*^b^*	Endosperm Genotypic Frequency*^c^*				χ^2^ Value (Probability)*^c^*	Allelic/Gametic Frequency*^d^*		
		*F_ddd_*	*F_Ddd_*	*F_DDd_*	*F_DDD_*		*F_d_*	*F_d’_*	*F_d’’_*
*SD_7-1_*									
Ex. ^#^1 (720, 0 DAR, 6%)	G (43, 100%)	0.256	0.256	0.256	0.232	0.07 (0.995)	0.512^ns^	0.512^ns^	0.511^ns^
Ex. ^#^2 (810, 3 DAR, 22%)	G (176, 98%)	0.256	0.216	0.307	0.222	3.68 (0.298)	0.517^ns^	0.563^ns^	0.472^ns^
Ex. ^#^3 (631, 10 DAR, 77%)	G (481, 99%)	0.262	0.247	0.249	0.241	0.44 (0.932)	0.510^ns^	0.511^ns^	0.509^ns^
	NG (138, 93%)	0.283	0.232	0.246	0.239	0.84 (0.839)	0.522^ns^	0.529^ns^	0.514^ns^
	G+NG (619)	0.267	0.244	0.249	0.241	0.99 (0.804)	0.513^ns^	0.515^ns^	0.511^ns^
*SD_1-2_*									
Ex. ^#^1 (1091, 1 DAR, 25%)	G (250, 92%)	0.376	0.296	0.200	0.128	35.4 (<0.0001)	0.624***	0.576*	0.672***
Ex. ^#^2 (1309, 10 DAR, 55%)	G (674, 93%)	0.261	0.236	0.242	0.261	0.09 (0.710)	0.500^ns^	0.497^ns^	0.503^ns^
Ex. ^#^3 (1096, 1 DAR, 24%)	G (280, 100%)	0.396	0.300	0.207	0.096	55.3 (<0.0001)	0.650***	0.604***	0.696***
	NG (801, 99%)	0.205	0.236	0.257	0.302	16.1 (0.0011)	0.451**	0.462*	0.441***
	G+NG (1081)	0.254	0.253	0.244	0.249	0.26 (0.967)	0.503^ns^	0.499^ns^	0.507^ns^
*SD_12_*									
Ex. ^#^1 (1980, 7 DAR, 20%)	G (381, 98%)	0.367	0.234	0.257	0.142	39.4 (<0.0001)	0.613***	0.625***	0.601***
Ex. ^#^2 (639, 10 DAR, 34%)	G (215, 99%)	0.381	0.195	0.228	0.195	20.4 (0.0001)	0.593*	0.609**	0.577*
Ex. ^#^3 (633, 14 DAR, 26%)	G (161, 99%)	0.372	0.186	0.298	0.143	21.2 (<0.0001)	0.615**	0.671***	0.559^ns^
	NG (234, 50%)	0.269	0.209	0.291	0.231	3.77 (0.286)	0.519^ns^	0.560^ns^	0.479^ns^
	G+NG (395)						0.544^ns^	0.588***	0.499^ns^
Ex. ^#^4 (a random sample of 484 seeds)		0.287	0.231	0.285	0.196	11.3 (0.0101)	0.545*	0.572**	0.519^ns^
*SD_7-1_* & *SD_12_*									
Ex. ^#^1 (1529, 14 DAR, 29%)	G (436, *SD_12_*)	0.477	0.181	0.216	0.126	127 (<0.0001)	0.675***	0.693***	0.661***
	G (436, *SD_7-1_*)	0.225	0.239	0.280	0.257	2.97 (0.396)	0.484^ns^	0.504^ns^	0.463^ns^
Ex. ^#^2 (1019, 35 DAR, 81%)	NG (121, *SD_12_*)	0.149	0.231	0.165	0.454	29 (<0.0001)	0.347***	0.314***	0.380**
	NG (121, *SD_7-1_*)	0.314	0.256	0.198	0.231	3.46 (0.326)	0.541^ns^	0.512^ns^	0.570^ns^

a Listed in the parentheses are the total number of seeds received the number of days of after-ripening (DAR) treatment before the germination test and the mean germination rate at the seventh day after imbibition.

b Listed in the parentheses are the number and proportion of genotyped seeds from the germinated (G) or nongerminated (NG) subpopulation.

c The letters *D* or *d* in subscripts represent dormancy-enhancing or -reducing alleles in endosperm genotypes, which were tested against the 0.25:0.25:0.25:0.25 expectation.

d The overall allelic frequency (*F_d_*) and the male (*F_d_*_’_) and female (*F_d’_*_’_) gamete frequencies for the *d* allele are defined in Table 2. The estimates for *SD_12_* in the joined population (G+NG in *SD_12_* Ex. ^#^3) are means weighted by the germination rate 26%. The superscripts indicate that the difference of the estimate from 0.5 was not significant at *P* = 0.05 (^ns^) or significant at *P* < 0.05 (*), <0.01(**), or <0.001 (***). The underlined estimates indicate *F_d’_*_’_>*F_d_*_’_ for *SD_1-2_* or *F_d_*_’_>*F_d’_*_’_ for *SD_12_* at the significance level of *P* < 0.05.

### *SD_7-1_* had neither endospermic nor embryonic effect on germination

**Figure 2:**
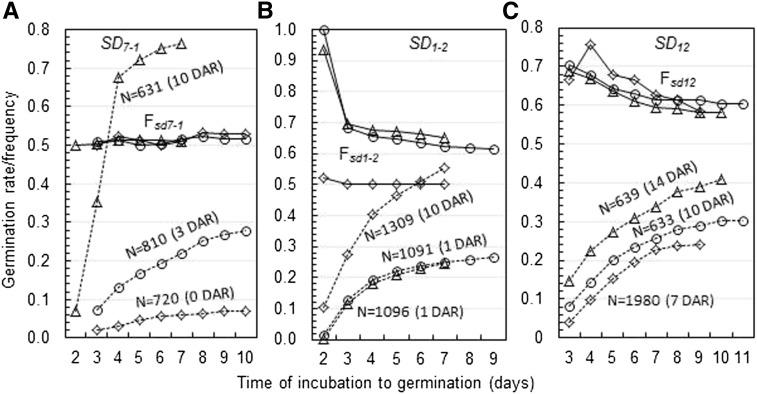
Germination and allelic frequency distributions in germinated subpopulations segregating for the seed dormancy loci *SD_7-1_* (A), *SD_1-2_* (B), and *SD_12_* (C). Dotted lines indicate germination distributions for three independent experiments (open diamonds, circles, and triangles), which were conducted for each locus using the indicated number (N) of F_2_ seeds received given days of after-ripening (DAR) treatment. Solid lines indicate frequency distributions for the dormancy-reducing alleles (F*_sd7-1_*, F*_sd1-2_*, or F*_sd12_*) in each of the germinated subpopulations. Note: the expected allelic frequency for the genetic equilibrium status is *p* = 0.5 and frequencies for the dormancy-enhancing alleles (1-*p*) are not shown.

Three germination experiments were conducted for 0-(Ex. ^#^1), 3-(Ex. ^#^2), or 10-(Ex. ^#^3) day after-ripened (DAR) seeds derived from the hybrid F_1_SD7-1_ (Table 1). Germination rate was 6% for Ex. ^#^1 and 22% for Ex. ^#^2, and germinated seeds were genotyped with the marker RID12. In the germinated subpopulations, four endosperm genotypes fit the expectation (Table 3), and frequencies for the dormancy-enhancing (*F_SD7-1_*) and -reducing (*F_sd7-1_*) alleles distributed around 0.5 during the germination period (Figure 2A). Thus, the selection for early germinated seeds did not alter the genetic equilibrium at *SD_7-1_*, and endosperm or embryo genotypic variation did not contribute to the phenotypic variation in germination velocity.

**Figure 3:**
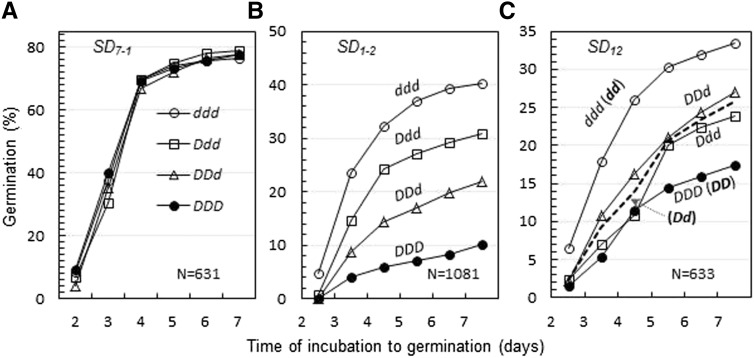
Cumulative germination distributions for four endosperm genotypes of seeds. Seeds were sampled from populations segregating for the seed dormancy locus *SD_7-1_* (A), *SD_1-2_* (B), or *SD_12_* (C). N was the number of seeds used for the germination experiment. Both germinated and nongerminated seeds were determined for endosperm genotypes, which are indicated by combinations of dormancy-enhancing (*D*) and/or -reducing (*d*) alleles. Embryo genotypes for *SD_12_* are listed in the parentheses (C).

Ex. ^#^3 yielded 77% germinated seeds and both germinated and nongerminated seeds were genotyped. Endosperm genotypic frequencies in the two subpopulations fit the expectation (Table 3), which confirmed the observations in Ex. ^#^1 and 2. In addition, the four genotypes of seeds were same in germination distribution pattern (Figure 3A), indicating that they were identical in the degree of seed dormancy. Because *SD_7-1_* had neither endospermic nor embryonic effect on germination in the three experiments, the dormancy gene should express in the maternal tissue(s) during seed development.

### *SD_1-2_* was associated with an endospermic effect on germination

Three germination experiments were conducted for 1-(Ex. ^#^1 & 3) and 10-(Ex. ^#^2) DAR seeds derived from the hybrid F_1_SD1-2_ (Table 1). Germination rate was 25% for Ex. ^#^1 and 55% for Ex. ^#^2 at the seventh day after imbibition, and germinated seeds were genotyped with the markers RM315 or RM3602. In the germinated subpopulation of Ex. ^#^1, endosperm genotypic frequencies dramatically deviated from the expectation, with *F_sd1-2sd1-2s1-2_* (0.38) >*F_SD1-2sd1-2sd1-2_* (0.30) > *F_SD1-2SD1-2sd1-2_* (0.20) > *F_SD1-2SD1-2SD1-2_* (0.13) (Table 3), and the frequency of the dormancy-reducing allele *F_sd1-2_* varied from 0.81 to 0.64 during the germination period from days 2 to 7 (Figure 2B). The genotypic segregation pattern and greater *F_sd1-2_* estimates in the germinated subpopulation indicate that *SD_1-2_* expressed in the offspring tissue. However, endosperm genotypic frequencies fit the 0.25:0.25:0.25:0.25 expectation (Table 3) and *F_sd1-2_* distributed around 0.5 during the germination period (Figure 2B) in the germinated subpopulation of Ex. ^#^2. Results from Ex. ^#^2 suggested that the inhibitory effect of *SD_1-2_* on germination was released during the after-ripening period of 10 d and seed dormancy left in the sample could be due to the other factors in the genetic background.

Ex. ^#^3 (24%) was a repeat of Ex. ^#^1 and both germinated and nongerminated seeds were genotyped for endosperms. Segregation distortion was observed in the germinated (*F_sd1-2_* = 0.65) and nongerminated (*F_sd1-2_* = 0.45) subpopulations, but not in their joined population (*F_sd1-2_* = 0.50, Table 3). In the germinated subpopulation, patterns for both genotypic (*F_sd1-2sd1-2s1-2_* > *F_SD1-2sd1-2sd1-2_* > *F_SD1-2SD1-2sd1-2_* > *F_SD1-2SD1-2SD1-2_*) and allelic (Figure 2B) frequency distributions were similar to those observed in Ex. ^#^1. In contrast, the nongerminated subpopulation displayed an opposite genotypic frequency distribution pattern (*F_sd1-2sd1-2s1-2_* < *F_SD1-2sd1-2sd1-2_* < *F_SD1-2SD1-2sd1-2_* < *F_SD1-2SD1-2SD1-2_*), with the allelic frequency in favor of the dormancy-enhancing allele (*F_SD1-2_* = 0.55). In addition, the four endosperm genotypes differed from each other in germination velocity, with *sd_1-2_sd_1-2_sd_1-2_* > *SD_1-2_sd_1-2_sd_1-2_* > *SD_1-2_SD_1-2_sd_1-2_* > *SD_1-2_SD_1-2_SD_1-2_* (Figure 3B). The distinct genotypic difference indicates that *SD_1-2_* regulates germinability through the endosperm tissue.

It is noted that gamete genotypic frequencies for *sd_1-2_*, which were estimated based on germinated subpopulations of Ex. ^#^1 and 3, were greater (*P* < 0.025) in the female (*F_sd1-2’_*_’_ = 0.68−0.69) than in the male (*F_sd1-2_*_’_ = 0.57−0.60) (Table 3). This result indicates that endosperm genotypes of the early germinated seeds consisted of more *sd_1-2_* allele donated from the female than from the male gametes. The difference between *F_sd1-2’_*_’_ and *F_sd1-2_*_’_ has nothing to do with allelic differentiation in gamete fertility or preferential fertilization, because gamete genotypic frequencies in the joined population of Ex. ^#^3 fit the Mendelian expectation (Table 3). In fact, the difference between *F_sd1-2’_*_’_ and *F_sd1-2_*_’_ is determined by frequencies for the two heterozygous endosperm genotypes (refer to formula in Table 2), which are different in the copy number (or dose) of the dormancy-reducing or -enhancing allele. In both of the germinated subpopulations, the frequency was greater for *SD_1-2_sd_1-2_sd_1-2_* (~0.3), which has two germination-promoting alleles from the female gametes, than for *SD_1-2_SD_1-2_sd_1-2_* (~0.2), which has one germination-promoting allele from the male gametes (Table 3). Therefore, the observed difference between *F_sd1-2’_*_’_ and *F_sd1-2_*_’_ can be accounted for by a dosage effect of the *sd_1-2_* (*SD_1-2_*) allele in endosperms on germination promotion (inhibition).

It was estimated based on model (4) for endosperm genotypes that the *SD_1-2_* locus consisted of only an additive effect on the time to germination (0.31 to 0.47 d) in the germinated subpopulations of Ex. ^#^1 and 3 (Table 4).

**Table 4:** Summary of estimated gene component effects of the *SD_1-2_* or *SD_12_* locus on the time period of incubation required for individual seeds to complete germination in germinated subpopulations*^a^*

Locus (Subpopulation)	Additive Effect, d	SE, d	*t*-Value	Probability	Model
*SD_1-2_* (*SD_1-2_* Ex. ^#^1)	0.47	0.08	5.76	<0.0001	Endosperm (4)
*SD_1-2_* (*SD_1-2_* Ex. ^#^2)	0.31	0.08	4.13	<0.0001	Endosperm (4)
*SD_12_* (*SD_12_* Ex. ^#^1)	0.22	0.11	2.08	0.0383	Embryo (3)
*SD_12_* (*SD_12_* Ex. ^#^2)	0.51	0.14	3.64	0.0003	Embryo (3)
*SD_12_* (*SD_12_* Ex. ^#^3)	0.53	0.22	2.41	0.0168	Embryo (3)
*SD_12_* (*SD_7-1_* & *SD_12_* Ex. ^#^1)	0.73	0.14	5.20	<0.0001	Embryo (3)

a Refer to Table 3 for additional information on the subpopulations. Gene component effects were estimated using the cited additive-dominance model for endosperm or embryo genotypes. Estimates for the component dominance effect are not listed as they are not significant at *P* = 0.05.

### *SD_12_* was associated with an embryonic effect on germination and a genetic differentiation in gamete preferential fertilization

Three germination experiments were conducted for 7-(Ex. ^#^1), 10-(Ex. ^#^3), or 14-(Ex. ^#^2) DAR seeds derived from the hybrid F_1_SD12_ (Table 1). Germination rate was 20% for Ex. ^#^1 and 36% for Ex. ^#^2 at the seventh day after imbibition, and the germinated seeds were genotyped with the marker SD12m13. In germinated subpopulations of the two experiments, endosperm genotypic frequencies dramatically deviated from the expectation (Table 3) and the frequency of the dormancy-reducing allele *F_sd12_* varied from 0.68 at day 3 to 0.60 at day 7 (Figure 2C). The *F_sd12_* estimates reduced gradually with the increase in incubation time in these two experiments, indicating that the genotypic variation partly contributed to the germination heterogeneity and the dormancy gene expressed in the offspring tissue.

Germination rate was 26% for Ex. ^#^3 at the seventh day after imbibition and both germinated and nongerminated (50%) seeds were genotyped. Segregation distortion for endosperm genotypes was observed in the germinated but not in the nongerminated subpopulation (Table 3) and the *F_sd12_* distribution pattern during the germination period was similar to those in the first two experiments (Figure 2C). In addition, the four endosperm genotypes of seeds displayed three germination distribution patterns, with the two heterozygotes (*SD_12_sd_12_sd_12_* and *SD_12_SD_12_sd_12_*) similar in germination velocity (Figure 3C). The association between germination distribution patterns and embryo genotypes is an indication that *SD_12_* is involved in embryo dormancy. It was estimated based on model (3) for embryo genotypes that the *SD_12_* locus consisted of only an additive effect on the time to germination (0.22−0.53 d) in the germinated subpopulations of Ex. ^#^1, 2, and 3 (Table 4).

It is noted that gamete genotypic frequencies for the dormancy-reducing allele *sd_12_* in the joined population of Ex. ^#^3 deviated from 0.5 for the male (*F_sd12_*_’_ = 0.588) but not for the female (*F_sd12’_*_’_ = 0.50) gametes (Table 3). The deviation suggests that there could be a SDL in the *SD_12_*-containing region affecting the fertilization of male gametes. To prove the hypothesis, a random sample of 484 F_2_ seeds was genotyped (Ex. ^#^4). Four endosperm genotypes in this sample also deviated from the expected equal frequency, with the allelic frequency in favor of *sd_12_* in the male (*F_sd12_*_’_ = 0.57), but not in the female (*F_sd12’_*_’_ = 0.52) gametes (Table 3). The genetic disequilibrium observed in the sizable random sample clearly indicates that the *SD_12_* locus was associated with a genetic differentiation in fertilization capability and male gametes with the dormancy-enhancing allele *SD_12_* tended to be less competitive in fertilization. The associated effect on gamete preferential fertilization contributed only part to the genetic disequilibrium in the germinated subpopulations, because the *F_sd12_*_’_ estimates (0.61-0.67) in the above-stated three experiments were numerically greater than that (0.57) in the random sample.

### Selection for early or late germination broke the genetic equilibrium for *SD_12_*, but not for *SD_7-1_*, in seed populations segregating for both loci

**Figure 4:**
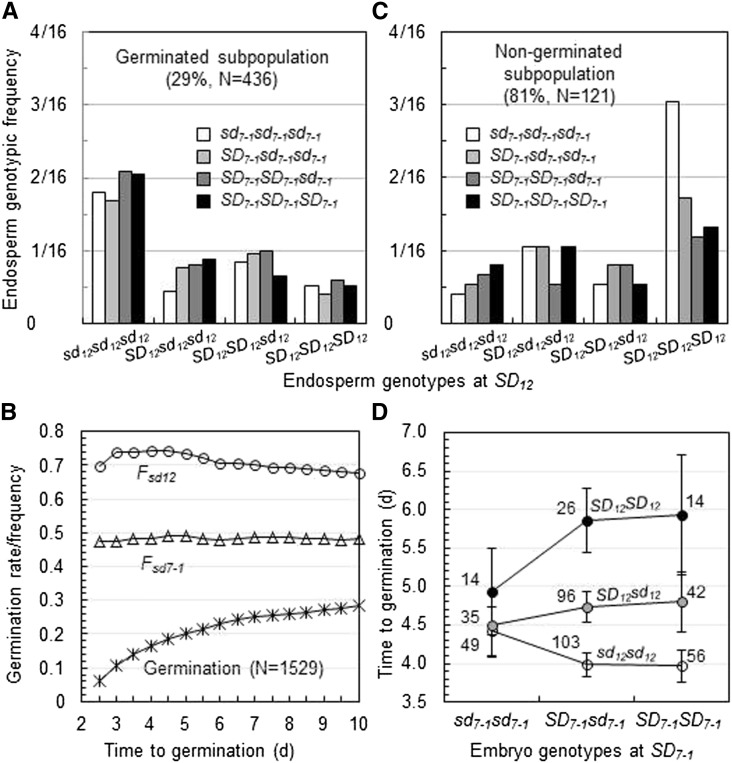
Genotypic or allelic frequency distributions in germinated and nongerminated subpopulations segregating for *SD_7-1_* and *SD_12_*. (A) and (C) Joint frequency distributions for endosperm genotypes of the two loci. The triploid genotypes for each of the two loci are represented by combinations of the dormancy-enhancing (upper case) and/or -reducing (low case) alleles. Refer to Table 3 (*SD_7-1_* and *SD_12_* Ex. ^#^1 and 2) for single-locus information of the germinated (A) and nongerminated (C) subpopulations. (B) Germination and allelic frequency distributions. N was the number of seeds used for the germination experiment. F*_sd7-1_* and F*_sd12_* are frequencies for the dormancy-reducing alleles at *SD_7-1_* and *SD_12_*, respectively, in the germinated subpopulation. (D) Genotypic differences in the time to germination. Data shown are means (circles), SEs (bars), and sample sizes (numbers) for the nine embryo genotypes of seeds.

Two germination experiments were conducted for 14-(Ex. ^#^1) or 35-(Ex. ^#^2) DAR seeds derived from the dihybrid F_1_SD7-1SD12_ (Table 1). Germination rate for the 14-DAR seed samples was 29%, which was lower than those for the 10- and 14-DAR seed samples segregating only for *SD_7-1_* (77%) and *SD_12_* (36%), respectively. The reduced germination rate indicates that pyramiding of the dormancy-enhancing alleles at *SD_7-1_* and *SD_12_* lengthened the dormancy duration. Germinated seeds in Ex. ^#^1 were genotyped for endosperms with the markers RID12 and SD12m13. In the germinated subpopulation, the joint frequency distribution for the two loci was dramatically biased in favor of the four genotypes homozygous (*sd_12_sd_12_sd_12_*) for the dormancy-reducing allele at *SD_12_*, the two (*SD_12_SD_12_sd_12_* and *SD_12_sd_12_sd_12_*) groups of genotypes heterozygous for the *SD_12_* locus were similar in frequency (Figure 4A), and allelic frequency distributions displayed two patterns, *i.e.*, *F_sd12_* varied from 0.74 to 0.68 whereas *F_sd7-1_* was constant around 0.5 during the germination period (Figure 4B), which were similar to those observed in the germinated subpopulations segregating only for the *SD_7-1_* or *SD_12_* locus (Figure 2, A and C). These results indicate that *SD_12_* played a major role in regulating germinability through the embryo, not endosperm tissue in the digenic system.

It appeared that *SD_7-1_* also interacted with *SD_12_* in embryos to influence germination. For example, the variation in the time to germination among the three *SD_12_* genotypes was smaller in the *sd_7-1_sd_7-1_* than in the *SD_7-1_sd_7-1_* or *SD_7-1_SD_7-1_* backgrounds in Ex. ^#^1 (Figure 4D). However, the two-way analysis of variance based on model (5) revealed that only the main effect of *SD_12_* was significant (see Supporting Information, Table S1). The main effect was contributed by the additive component of the *SD_12_* locus, based on model (3) for embryo genotypes (Table 4).

Germination rate was 81% for Ex. ^#^2 and nongerminated seeds were identified for endosperm genotypes at *SD_7-1_* and *SD_12_*. A relatively high selection strength (as indicated by the germination rate) for nongerminated seeds in this experiment is expected to suppress less dormant genotypes in the subpopulation. As expected, the joint frequency distribution for *SD_7-1_* and *SD_12_* was biased in favor of the four genotypes that are homozygous (*SD_12_SD_12_SD_12_*) for the dormancy-enhancing allele at *SD_12_*, in particular the digenic genotype (*sd_7-1_sd_7-1_sd_7-1_SD_12_SD_12_SD_12_*) (Figure 4C). Segregation distortion was detected for *SD_12_* but not for *SD_7-1_*, with the *F_sd12_* estimate (0.35) lower than the Mendelian expectation (0.5) (Table 3). The results from the nongerminated subpopulation support that the *SD_12_*-controlled embryo dormancy played a major role in inhibiting germination and also suggest that *SD_7-1_* may influence embryo dormancy as well in the digenic system, as implied by the observation in the germinated subpopulation of Ex. ^#^1 (Figure 4D).

In addition, a numerical difference in genotypic frequency for the *SD_12_* locus between male and female gametes, as estimated based on the nongerminated subpopulation, was observed (*F_sd12__’_* < *F_sd12’_*_’_ or *F_SD12_*_’_ > *F_SD12’_*_’_), which was opposite to the observation in the germinated subpopulation of Ex. ^#^1 (*F_sd12_*_’_ > *F_sd12’_*_’_) (Table 3). The observations in the digenic system also confirmed that the associated effect of *SD_12_* on male gamete preferential fertilization contributed only part to the genetic disequilibrium in the subpopulations segregating for the major seed dormancy locus.

## Discussion

### Applications of the endosperm genotype-based genetic approach

An endosperm genotype-based genetic approach was developed to determine whether previously mapped seed dormancy genes/QTL regulate germination through the embryo, endosperm or maternal tissues. This approach involves techniques: 1) partially after-ripening a segregating population of seeds to separate relatively less dormant from more dormant genotypes by germination testing, 2) sampling endospermic DNAs from individual germinated and nongerminated seeds, and 3) identifying all endosperm genotypes using regular PCR-based codominant markers. After DNA sampling, the newly germinated seeds can be selected to develop progeny lines. Thus, the techniques, similar to those reported for pregermination screening of genotypes (Chunwongse *et al.* 1993; Kang *et al.* 1998), can be also used for early selection of QTL alleles in breeding programs. Information collected using this approach includes: 1) endosperm and embryo genotypic frequencies for tested loci in germinated and nongerminated subpopulations (Table 3); 2) association between incubation times required for individual seeds to complete germination and allelic frequencies in the germinated subpopulation (the association was more informative before the seventh day, as shown in Figures 2, B and C); 3) genotypic differences for the degree of dormancy in a segregating population of seeds (Figure 4); 4) the dosage effect for a dormancy gene with an additive effect on germination in endosperms; and 5) genotypic frequencies for both male and female gametes that were involved in the double fertilization to form the seed sample. SDLs were frequently reported for mapping populations (Xu *et al.* 1997; Faris *et al.* 1998); the endosperm genotype-based genetic approach also can be used to characterize SDLs and artificial mutants for functions in gamete development and fertilization.

The genetic approach demonstrated in rice can be extended to the other cereal crops to characterize tissue-specific functions of mapped genes/QTL controlling seed dormancy and some other seed traits. Before DNA markers became prevalent for QTL mapping, Robertson (1952) combined morphological markers with an A-B (normal-supernumerary chromosome) translocation system to determine the tissue-specific function of the maize *viviparous-5* mutant. The mutant distinguishes itself from the wild type by white endosperm and albino embryonic leaves and was concluded to regulate germination through the embryo, not the endosperm tissue. The A-B system was used in the reported research to help separate the four endosperm genotypes, because the morphological markers are dominant and have only two phenotypes for a locus. Nowadays, many codominant DNA markers can be selected to genotype endosperms in many species. We also tried a quantitative real-time PCR method to genotype endosperms with a dominant DNA marker in the *SD_1-2_* region (data not shown) and found out that genotyping with regular PCR for a codominant marker was more reliable and cost-effective.

Compared with the endosperm genotype-based genetic approach, the embryo genotype-based genetic approach (Gu *et al.* 2008) provides less information, but it is relatively easy to conduct and can be used to distinguish an embryo from a maternal tissue effect on germination for a dormancy gene. If a seed dormancy gene functions in both maternal and offspring tissues, only the inhibitory effect on germination expressed in the offspring tissue(s) can be detected by the endosperm or embryo genotype-based genetic approach, because F_2_ seeds from single F_1_ plants do not segregate for the maternal tissue genotype (Table 2). For this case, a joint maternal-offspring model (*e.g.*, Zhang *et al.* 2004) could be used to estimate effects of a seed dormancy gene on germination through different component tissues.

### Genetic controls for types of seed dormancy

Natural variation for such a complex, adaptive trait as seed dormancy involves multiple genetic controls in different seed component tissues. This research provided evidence that *SD_12_*, *SD_1-2_*, and *SD_7-1_* are involved in the genetic controls of seed dormancy through the embryo, endosperm, and maternal tissues, respectively. *SD_12_* has the largest effect on seed dormancy in rice and its allele from SS18-2 delays germination (Gu *et al.* 2004). The major effect of *SD_12_* on germination was contributed mainly by the additive component in embryos (Table 4) and contributed to the germination heterogeneity of seeds on the heterozygous (*SD_12_sd_12_*) plants. Therefore, marker-assisted selection for *SD_12_* alleles can be conducted before (or after) germination using the endosperm (or embryo) genotype-based genetic approach, and phenotypic selection for an early or late germinated subpopulation from a segregating population of partially after-ripened seeds can be used to enrich the dormancy-reducing or -enhancing allele. *SD_12_* also was associated with the preferential fertilization of male gametes on the heterozygous plants. The associated effect is defined as the preferential fertilization, not gamete fertility, because plants homozygous or heterozygous for the *SD_12_* dormancy-enhancing allele had a normal seed setting rate (>95%) in this and previous research (Gu *et al.* 2004, 2008). Both the major effect on embryo dormancy and the association with gamete preferential fertilization must have restrained the distribution of the dormancy-enhancing allele during rice domestication. This also explains why the *SD_12_* locus was not detected in the other mapping populations reported so far. Further research will elucidate if the association arises from a pleiotropic effect of *SD_12_* or a gene linked to *SD_12_*.

*SD_1-2_* could be the first naturally occurring gene that has been unambiguously associated with endosperm-imposed dormancy. Two analytic strategies were used to recognize *SD_1-2_*’s endospermic effect on germination: 1) a distinct difference in germination rate among the four endosperm genotypes in a segregating population of partially after-ripened seeds (Figure 3B), and 2) a significant difference in genotypic frequency between female and male gametes estimated based on the germinated subpopulation (*F_sd1-2’_*_’_ > *F_sd1-2_*_’_ in Table 3). It appeared that the inhibitory effect of *SD_1-2_* on germination lasts only for several days after seed maturation or harvest. The previous research delimited *SD_1-2_* to a short genomic region encompassing the gene *Sd1* (or *GA20ox-2*) (Table 1; Ye *et al.* 2013). *GA20ox-2* encodes GA20-OXIDASE2 catalyzing the second-to-last steps of the gibberellin (GA) biosynthesis and the loss-of-functional mutation (*sd1*) reduces the GA level in vegetative tissues and plant height (Ashikari *et al.* 2002; Monna *et al.* 2002; Spielmeyer *et al.* 2002). The dormancy-enhancing (*SD_1-2_*) and -reducing (*sd_1-2_*) alleles at the *SD_1-2_* QTL couple with the GA functional and loss-of-functional alleles, respectively, and *SD_1-2_*’s effect on inhibiting germination could be compensated by GA application (Ye *et al.* 2013). The endosperm aleuronic cell layer is known to plays a central role in GA signaling to synthesize hydrolytic enzymes for cell wall weakening and food reserve mobilization during and post germination (Steber 2007). It is likely that *GA20ox-2* is the QTL underlying gene. Research is being conducted to prove the hypothesis and to identify mechanisms for dormancy development and germination by *SD_1-2_* expressed in endosperms.

*SD_7-1_* was confirmed to control maternal tissue-imposed dormancy using the perfect isogenic system (IL_SD7-1_ is heterozygous only for a 2-kb *SD_7-1_* intragenic segment, Table 1). The *SD_7-1_* functional allele was isolated from the “red” weedy rice line SS18-2 and encodes a transcription factor that promotes the abscisic acid biosynthesis in early development seeds to induce primary dormancy and also activates the flavonoid biosynthesis pathway in the lower epidermal cell layer of the pericarp tissue to produce red pigments (Gu *et al.* 2011). Because an endospermic or embryonic effect on germination was not detected in the isogenic background (Figures 2A and Figure 3A), the *SD_7-1_*-regulated dormancy-inducing events must also occur in the maternal tissue. *SD_7-1_* and *SD_12_* control seed dormancy through different tissues, but their effects on delaying germination is cumulative, because it took a longer after-ripening period for seeds from the digenic system (F_1_SD7-1SD12_ plants) to reach similar germination rates than seeds from the monogenic systems (F_1_SD7-1_ and F_1_SD12_ plants). Questions to be addressed include how genes functioning in different tissues work in the same genetic system to lengthen the dormancy duration, and if a gene controlling coat-imposed dormancy is also involved in embryo dormancy, and *vice versa*.

## Supplementary Material

Supporting Information
