# Supplementary material for: Genotyping of Endosperms to Determine Seed Dormancy Genes Regulating Germination Through Embryonic, Endospermic, or Maternal Tissues in Rice
Source: G3 (Bethesda). 2014 Dec 4;5(2):183–93. doi: 10.1534/g3.114.015362 (PMC4321027; doi:10.1534/g3.114.015362)
Supplement: Supporting Information [file supp_5_2_183__index.html]

Genotyping of Endosperms to Determine Seed Dormancy Genes Regulating Germination Through Embryonic, Endospermic, or Maternal Tissues in Rice — Supporting Information 

# Genotyping of Endosperms to Determine Seed Dormancy Genes Regulating Germination Through Embryonic, Endospermic, or Maternal Tissues in Rice

## Supporting Information for Gu *et al.*, 2015

**Files in this Data Supplement:**

- Table S1 - Analysis of variance for the time period of incubation (d) required for individual seeds to germinate in the germinated subpopulation segregating for the *SD7-1* and *SD12* loci. (PDF, 120 KB)
