# Supplementary material for: Genotyping of Endosperms to Determine Seed Dormancy Genes Regulating Germination Through Embryonic, Endospermic, or Maternal Tissues in Rice
Source: G3 (Bethesda). 2014 Dec 4;5(2):183–93. doi: 10.1534/g3.114.015362 (PMC4321027; doi:10.1534/g3.114.015362)
Supplement: Supporting Information [file supp_g3.114.015362_015362SI.pdf]

G3: Genes | Genomes | Genetics

Genotyping of Endosperms to Determine Seed Dormancy Genes Regulating Germination through Embryonic, Endospermic or Maternal Tissues in Rice

Xing-You Gu[1], Jinfeng Zhang[2], Heng Ye, Lihua Zhang, and Jiuhuan Feng

Plant Science Department, South Dakota State University, Brookings, SD 57007
[2]Present address: Plant Gene Engineering Lab, DBN Agr. Sci&Tech Research Institute, Beijing 100085, China

[1]Corresponding author: Xing-You Gu; mailing address: Box 2140C, Brookings, SD 57007, USA; phone: +1(605) 688-6908; fax: +1(605) 688-4452; email: Xingyou.gu@sdstate.edu

DOI: 10.1534/g3.114.015362

Table S1 Analysis of variance for the time period of incubation (d) required for individual seeds to germinate in the germinated subpopulation segregating for the *SD_{7-1}* and *SD_{12}* loci (refer to "*SD_{7-1}* & *SD_{12}* Ex. [#]1" in Table 3 for additional information)

Source of variance	D.F.	SS	MS	F-value	Probability
SD_{12}	2	88.28	44.141	10.61	<0.0001
SD_{7-1}	2	3.77	1.887	0.45	0.63
SD_{12}×SD_{7-1}	4	18.63	4.657	1.12	0.34
Error	427	1777.1	4.162		
**Total**	**435**	**1910**			
